# Supplementary material for: Persistent activity of aerobic methane-oxidizing bacteria in anoxic lake waters due to metabolic versatility
Source: Nat Commun. 2024 Jun 21;15:5293. doi: 10.1038/s41467-024-49602-5 (PMC11192741; doi:10.1038/s41467-024-49602-5)
Supplement: Supplementary file 1 — Supplementary Information [file 41467_2024_49602_MOESM1_ESM.pdf]

Supplementary Information

Persistent activity of aerobic methane-oxidizing bacteria in anoxic lake waters due to metabolic versatility

Authors:

Sina Schorn [1,5*], Jon S. Graf [1], Sten Littmann [1], Philipp F. Hach [1], Gaute Lavik [1], Daan R. Speth [1,2], Carsten J. Schubert [3,4], Marcel M. M. Kuypers [1], and Jana Milucka [1]

Affiliations:

[1] Max Planck Institute for Marine Microbiology, Bremen, Germany

[2] Division of Microbial Ecology, Center for Microbiology and Environmental Systems Science, University of Vienna, Vienna, Austria

[3] Swiss Federal Institute of Aquatic Science and Technology (Eawag), Kastanienbaum, Switzerland

[4] Swiss Federal Institute of Technology, Institute of Biogeochemistry and Pollutant Dynamics, Zürich, Switzerland

[5] Present address: Department of Marine Sciences, University of Gothenburg, Gothenburg, Sweden

* For correspondence: sina.schorn@gu.se

Supplementary Notes

Note 1: Bulk methane carbon assimilation rates in 2018 and 2019

The same incubation experiments as in September 2017 were conducted with water samples collected in October 2018 and May 2019. Also in both later campaigns, three incubation depths were chosen to encompass the oxic-anoxic interface and two anoxic depths. However, it should be noted that the absolute depths of these incubations varied between the campaigns, as the depth of the oxycline varied between 121 m in 2017, 160 m in 2018 and 175 m in 2019 (Figure S1). In any case, methane

oxidation to CO_2 exceeded assimilation into biomass at all depths in October 2018 as well as in May 2019. However, we still observed high assimilation rates ranging from 25% to 48% in October 2018 and from 23 to 33% in May 2019 across all depths (Table S1).

Note 2: Presence of putative *Methylobacter*-like gamma-MOB in Lake Zug

From the three sampled depths, 14 metagenome-assembled bins were obtained which contained the *pmoA* gene. Of those, 13 bins belonged to gamma-MOB of the Methylococcales order, more specifically to genera SXIZ01, *Methylobacter* A, KS41, *Methylovulum*, UBA4132, and UBA10906; and one bin belonged to *Candidatus* Methylomirabilis limnetica. The phylogeny of the bins was inferred from concatenated marker genes, as none of the bins contained a 16S rRNA gene. However, we also recovered ten 16S rRNA genes belonging to gamma-MOB of the Methylococcales order from the metagenomes (2 sequences from 123 m, 3 from 135 m, and 5 from 160 m). The recovered 16S rRNA gene sequences closely affiliated with genera *Methylobacter*, KS41, uncultured *Crenothrix*, uncultured *Methyloglobulus*, UBA10906, UBA4132 and other uncultured Methylomonadaceae (Supplementary Dataset 1). Thus, although no 16S rRNA gene sequences were recovered from the gamma-MOB bins, we observed consistency between the taxonomic affiliation of the retrieved 16S rRNA gene sequences and the metagenomic bins.

One of our recovered 16S rRNA gene sequences was closely related to *Methylobacter tundripaludum*. The type strain of *M. tundripaludum* (strain SV96^T) has been characterized by straight, rod-shaped cells with lengths of up to 2.5 µm [1], highly reminiscent of the large rod-shaped MOB described in our study. A similar cell morphology has been reported for a *Methylobacter* isolate obtained from the water column of Lacamas Lake [2]. While rod-shaped cells appear to be the predominant type among *Methylobacter* species, coccoid morphologies have been documented as well [3].

To further confirm the taxonomic affiliation of the rods, we used a CARD FISH probe that was designed to specifically target *Methylobacter* species. This *Methylobacter*-specific FISH probe (probe MLB482 [4]) indeed hybridized the large rod-shaped MOB in our samples. However, it should be noted that also other MOB cell types were stained by the probe and overall there was no large difference between the hybridization pattern of the probe MLB482 compared to probe mix Mγ84+Mγ704, known to target type I methanotrophs. In any case, the characteristic cell shapes of the large rods alongside our molecular data suggest that the active MOB in our anoxic incubations may in fact be a *Methylobacter*-like species, in agreement with frequent observations of MOB of this genus in anoxic environments [5-8].

On the other hand, coccoid gamma-MOB cell types observed in our incubations could be related to *Methylovulum*, as cultured members of this genus are known as obligate aerobic methane-oxidizers with a distinctive coccoid cell morphology [9,10]. In environmental settings, *Methylovulum* methanotrophs have been observed primarily at and below oxic-anoxic interfaces [11,12].

Note 3: Contribution of other bacterial methanotrophs to methane oxidation in Lake Zug

In previous studies it was shown that gammaproteobacterial methane-oxidizing bacteria consume a large portion of the upwards-diffusing methane near the oxycline and also in the anoxic hypolimnion of Lake Zug [13]. Among others, filamentous bacteria of the genus *Crenothrix* were shown to contribute to this methane removal [14], mainly at the oxic-anoxic interface but also under anoxic, denitrifying conditions. In agreement with these observations we also detected active *Crenothrix*-like MOB in our hypoxic as well as anoxic incubations. The *Crenothrix*-like filaments assimilated methane-derived carbon, even though their activity in our anoxic nitrate-supplemented incubations seemed more sporadic as compared to the large rod-shaped *Methylobacter*-like MOB. Out of the 31 measured putative *Crenothrix* sp. cells, three were comparably high enriched as the putative *Methylobacter* cells (i.e. large rods). However, these three cells belonged to the same *Crenothrix* sp. filament. As the abundance of *Crenothrix*-like cells in our incubations

was generally much lower than that of the other gamma-MOB groups, the sporadically enriched filamentous cells were not included in further calculations of single cell methane oxidation rates. It should be noted that *Crenothrix*-like MOB were solely identified based on their conspicuous filamentous cell shapes; it is therefore possible that not all of the measured filaments were indeed *Crenothrix*.

Furthermore, NC10 bacteria belonging to *Candidatus* Methylomirabilis limnetica have been observed to bloom to high abundances in the anoxic hypolimnion of Lake Zug [15]. This bloom coincided with the presence of nitrogen oxides in the anoxic bottom waters, which could have stimulated their growth under these specific conditions. The contribution of NC10 bacteria to methane oxidation was not quantified in the previous study. Our data now suggest that NC10 bacteria did not contribute significantly to the retention of methane carbon in the hypolimnion as the assimilation of ^{13}C-methane-derived carbon into their biomass was negligible compared to the other gamma-MOB groups (Figure S7). The low ^{13}C enrichment observed for NC10 bacteria as well as for small rod-shaped and coccoid MOB could also originate from cross-feeding on methane-derived compounds, rather than direct methane carbon assimilation.

Note 4: Possible reduction in growth rates due to grazing and viral infections

The ^{13}C-based growth rates did not seem to match the increase in cell numbers in our hypoxic and anoxic incubations for the large rod-shaped MOB (Table S2). We hypothesize that this discrepancy might be explained by the higher precision of quantifying ^{13}C uptake vs. counting individual cells, as well as a reduction in cell numbers due to predation, as in fact, an anaerobic ciliate dwelling in the anoxic hypolimnion of Lake Zug might prey on the gamma-MOB [16]. Due to their size, MOB could be attractive prey for various predatory protists [17]. Additionally, viral lysis might also reduce the population size of the gamma-MOB [18].

Note 5: Sources of oxygen in incubations and in situ

All incubation experiments were set up within an anaerobic hood and performed in sealed serum bottles in order to minimize potential oxygen contamination during the incubation process. However, based on our experimental setups (ex situ bottle incubations), we cannot completely eliminate the possibility of trace amounts of oxygen being present in the incubations below the detection range of our optodes (low nanomolar). Our lowest detected anaerobic methane oxidation rates were about 60 nM d^{-1}, for which - depending on growth stage - around 120 nM O_2 d^{-1}, or at least 70 nM O_2 d^{-1} would be needed, considering a ratio of 1:2 and 1:1.2 for CH_4 and O_2 (over a duration of 8 days, a total of ca. 0. 96 µM or 0.5 µM O_2, would be needed, respectively) [19]. Although we did not detect such concentrations of oxygen in our incubations at any measured time point, we cannot exclude that oxygen concentrations below the detection limit of our sensor were periodically present. To date, all proposed scenarios for gamma-MOB growth under anoxic conditions invoke the presence of trace amounts of oxygen for initial methane activation to methanol, and it is possible that our incubation conditions provide that.

In the environment, anoxic waters are known to periodically receive oxygen inputs, for example through oxygen intrusions from the oxygenated waters. With increasing distance from the oxycline, such oxygen transport could be reduced thus affecting MOB activity eventually, despite high methane and nitrate concentrations in deep, anoxic waters. In addition, biological sources have been proposed to generate oxygen in anoxic waters, including dark oxygen production by NC10 bacteria or ammonia-oxidizing archaea (AOA). Both organisms produce oxygen under dark, anoxic conditions through the dismutation of nitric oxide [20,21]. Although it is assumed that the oxygen produced by these processes primarily serves to maintain their own aerobic metabolism, any oxygen that may escape from the cells could become available for other bacteria as well. We detected sequences belonging to *Nitrosopumilus*, an abundant genus of AOA, in our metagenomics datasets, albeit at low relative abundances particularly

in the deepest depth, comprising 2.1% at 123 m, 0.8% at 135 m, and 0.4% at 160 m (Supplementary Dataset 4).

Additionally, it has been proposed that O_2 generated during methanobactin-mediated metal ion reduction can be used for methane oxidation by *Methylosinus trichosporium,* an alphaproteobacterial MOB [22]. However, its relevance in Lake Zug remains uncertain due to the lower abundance of alpha-MOB in the anoxic bottom waters. The gamma-MOB examined in this study lacked the gene set for methanobactin biosynthesis (i.e. Mbn operon).

Note 6: Calculation of single cell methane oxidation rates of gamma-MOB and comparison to anaerobic archaeal and bacterial methanotrophs

To calculate single cell methane oxidation rates we divided the bulk methane oxidation rates by the CARD FISH-based gamma-MOB cell counts at various time points (see Table S3). For incubations performed under hypoxic conditions, we divided the bulk rate by the sum of all gamma-MOB, because our nanoSIMS data showed that all gamma-MOB groups were almost equally active. For single cell methane oxidation rates in anoxic incubations, we divided the bulk rate by the sum of only the large rod-shaped gamma-MOB, as nanoSIMS showed that only this morphotype was consistently active in our anoxic incubations. However, there are some uncertainties associated with the calculation of the single cell methane oxidation rates, specifically for the hypoxic incubations. The bulk rate was divided by the total number of gamma-MOB in the incubation, although the active gamma-MOB population consisted of four morphologically distinct groups characterized by differences in cell size and nanoSIMS-based activity, so the actual methane oxidation rate of individual cells might differ. The rates calculated for the three time points in hypoxic incubations were indeed somewhat variable, which might reflect the diversity of active MOB. In comparison, in anoxic incubations, only the large rods, and to a lesser extent the filamentous cells, showed pronounced activity, so for calculation of single cell methane oxidation rates,

only the cell numbers of large rods were considered, minimizing the variability as compared to hypoxic incubations. Indeed, the anaerobic single cell methane oxidation rates were more reproducible across the different time points (Table S3). It should be noted that the NC10 bacteria did not show significant ^{13}C assimilation and their contribution to overall methane oxidation therefore could not be determined.

Under anoxic conditions, the per cell methane oxidation rate of the large rod-shaped gamma-MOB amounted to 9.1 fmol CH_4 cell^{-1} day^{-1}. This rate exceeds reported per cell rates of known anaerobic archaeal and bacterial methanotrophs (ANME groups and NC10) performing either nitrite-dependent AOM (0.4 fmol CH_4 cell^{-1} day^{-1}) [23], or sulfate-dependent AOM (0.7 fmol CH_4 cell^{-1} day^{-1}) [24], as well as single cell rates of nitrite-dependent methane-oxidizing NC10 bacteria (up to 0.4 fmol CH_4 cell^{-1} day^{-1}) [25]. Also the assimilation rate of methane carbon into cellular biomass of the large rod-shaped gamma-MOB (8.6 fmol ^{13}C cell^{-1} d^{-1}) was orders of magnitude higher than C assimilation rates reported for anaerobic ANME-2 archaea (0.39 amol C cell^{-1} day^{-1}) [26], even though it should be kept in mind that methane carbon likely constitutes only a minor source for biomass for ANME archaea. The higher per cell rates of the Lake Zug gamma-MOB can be largely explained by their comparably large cell size ranging from 0.9 µm^3 to 7.4 µm^3 [14,27] (Table S2). This far exceeds the size of other methanotrophs such as ANME archaea (0.07 µm^3) [28] and NC10 bacteria (0.05 to 0.2 µm^3) [25].

Our per cell rates compare well to ammonium oxidation rates by aerobic ammonium-oxidizing archaea (AOA) (1 to 8 fmol N cell^{-1} day^{-1}) [29] which is interesting given the similarity between the methane monooxygenase, used by MOB to oxidize methane, and the ammonium monooxygenase, used by AOA to oxidize ammonium. Interestingly, also aerobic ammonium oxidizers (as well as nitrite oxidizers) show activity at low, nanomolar oxygen concentrations, and it has been proposed that their in situ activity at oxic-anoxic interfaces or at anoxic depths is likely maintained by irregular oxygen intrusions [30].

Supplementary Figures

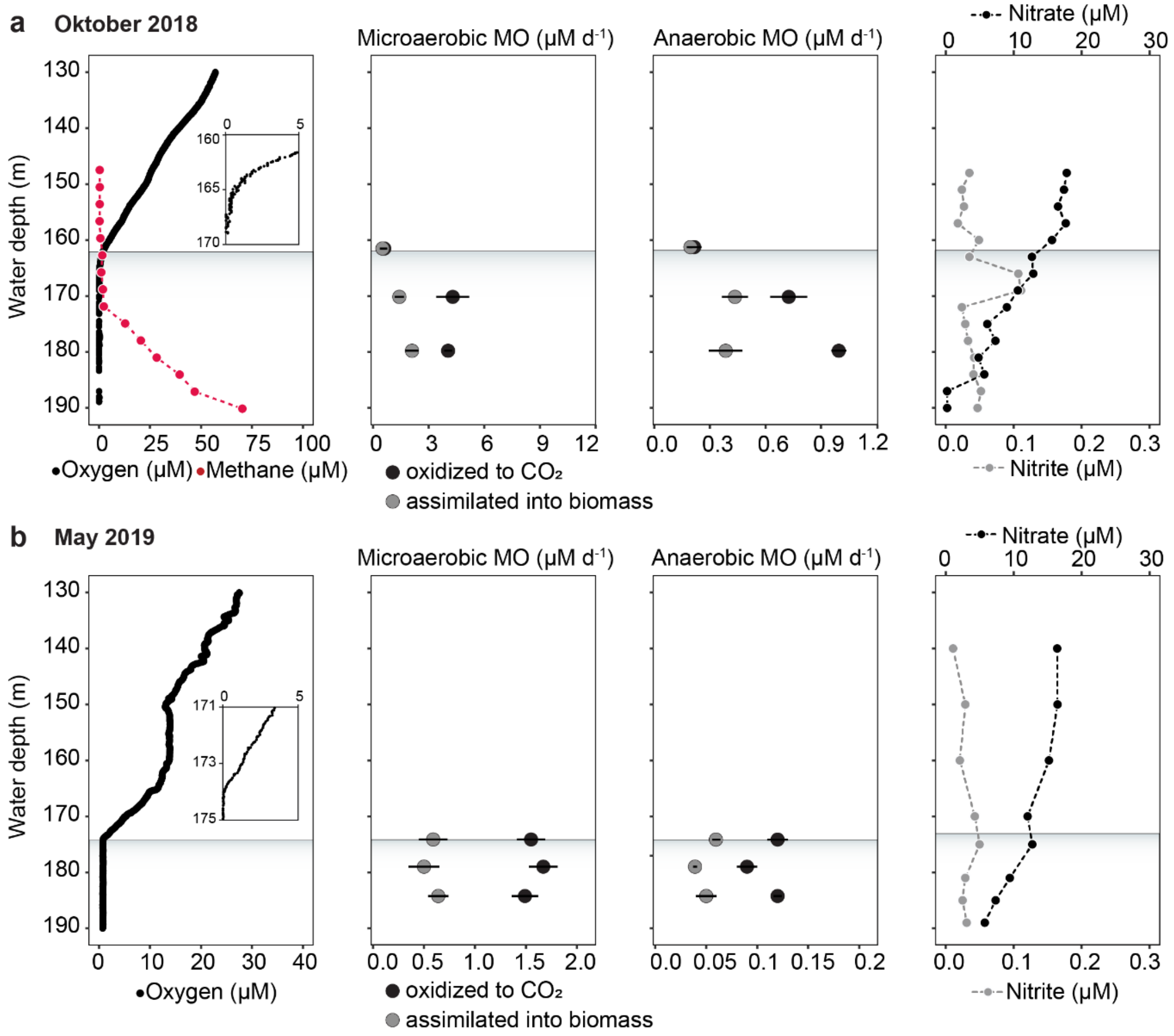

Figure S1. Lake Zug water column profiles recorded in October 2018 (a) and May 2019 (b). In situ concentration profiles of oxygen (black) and methane (red, only for October 2018) below 130 m water depth. Inlets show oxygen concentrations at the oxic-anoxic interface (panel 1). Rates of microaerobic and anaerobic methane oxidation (MO) to carbon dioxide (black symbols) and carbon assimilation to biomass (gray symbols; panel 2 and 3). Note that rates of anaerobic methane oxidation are approximately 10-fold lower than those of microaerobic methane oxidation. In situ concentrations of nitrate and nitrite (black and gray symbols, respectively) (panel 4). Statistically significant methane oxidation and assimilation rates (one-sided t-test, $p < 0.05$) are presented as mean values ± SEM calculated from the linear regression of the first five time points across 8 days of incubation. Source data are provided as a Source Data file.

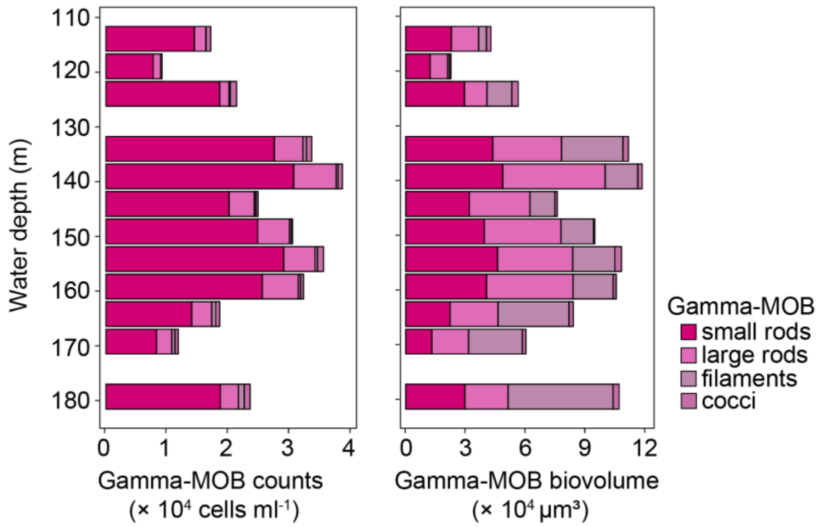

Figure S2. Total abundance and population biovolume of gamma-MOB in the hypolimnion of Lake Zug. Gamma-MOB cells were quantified using CARD FISH-based cell counts. The biovolume of distinct gamma-MOB groups was calculated by multiplying their cell counts per depth by their individual cell volume. Source data are provided as a Source Data file.

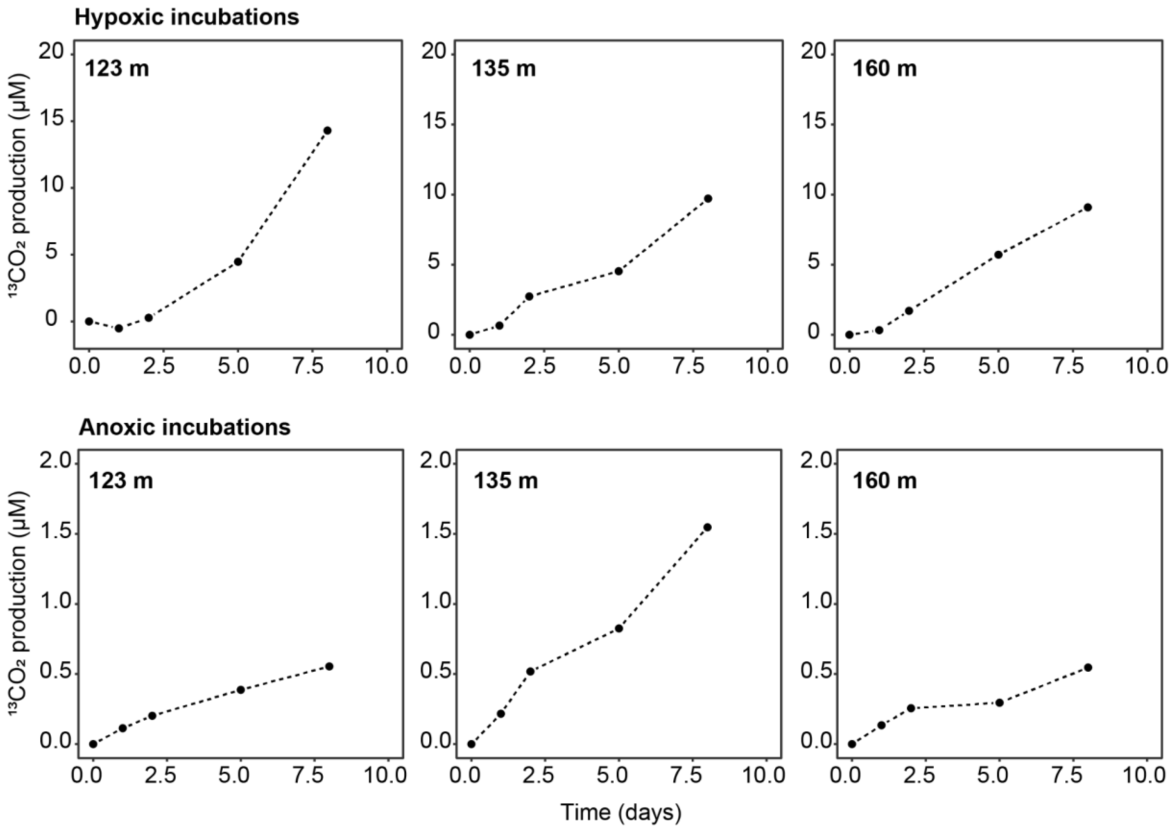

Figure S3. Time course of $^{13}CO_2$ production in hypoxic and anoxic incubations across 8 days. Source data are provided as a Source Data file.

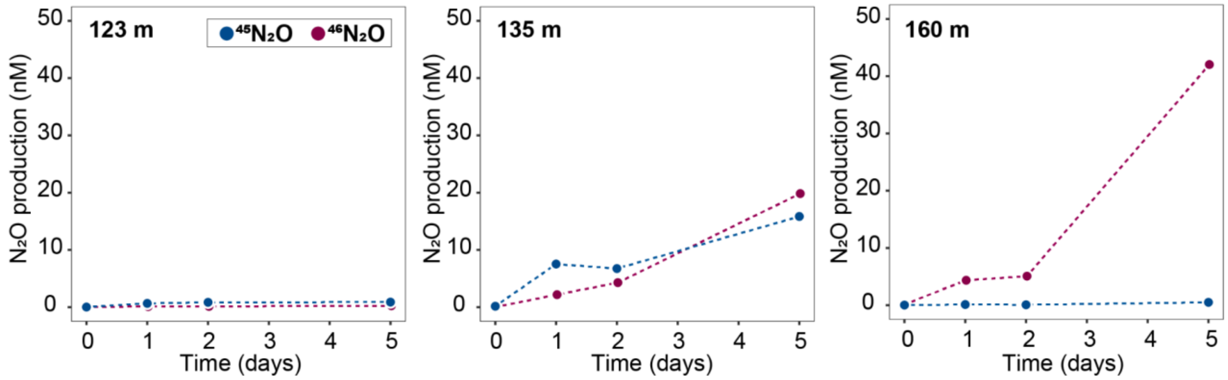

Figure S4. N_2O production in anoxic ^{15}N-nitrate-amended incubations. Shown is the production of ^{15}N-labeled N_2O both as $^{45}N_2O$ (blue) and $^{46}N_2O$ (red) across the first 5 days of incubation. Source data are provided as a Source Data file.

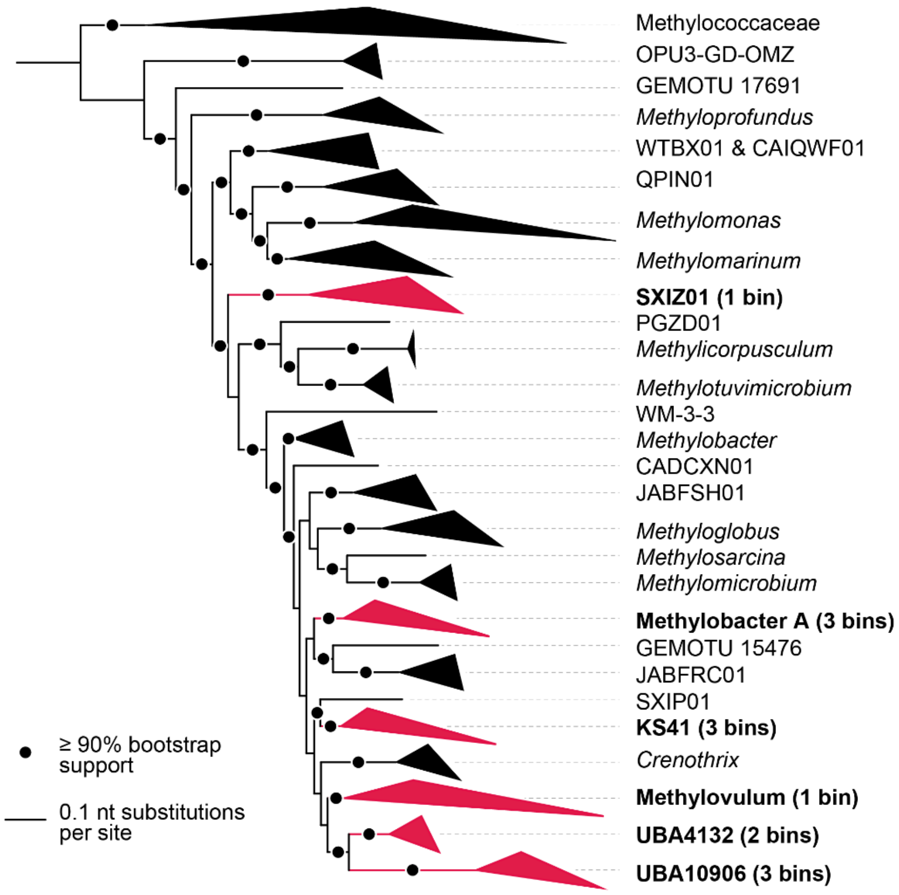

Figure S5. Genome phylogeny of Lake Zug gamma-MOB. The genome tree represents the phylogenetic relationships of the Methylococcales order, based on the concatenation of marker genes from selected genera, including bins recovered from Lake Zug metagenomes containing the *pmoA* gene. Genera affiliated with the Lake Zug bins are visually highlighted in bold and marked in red. The number of bins per genus is indicated within brackets. The robustness of the tree is presented by circles on branches that received 90% or more bootstrap support. The taxonomic classification of the bins was inferred using the GTDB database.

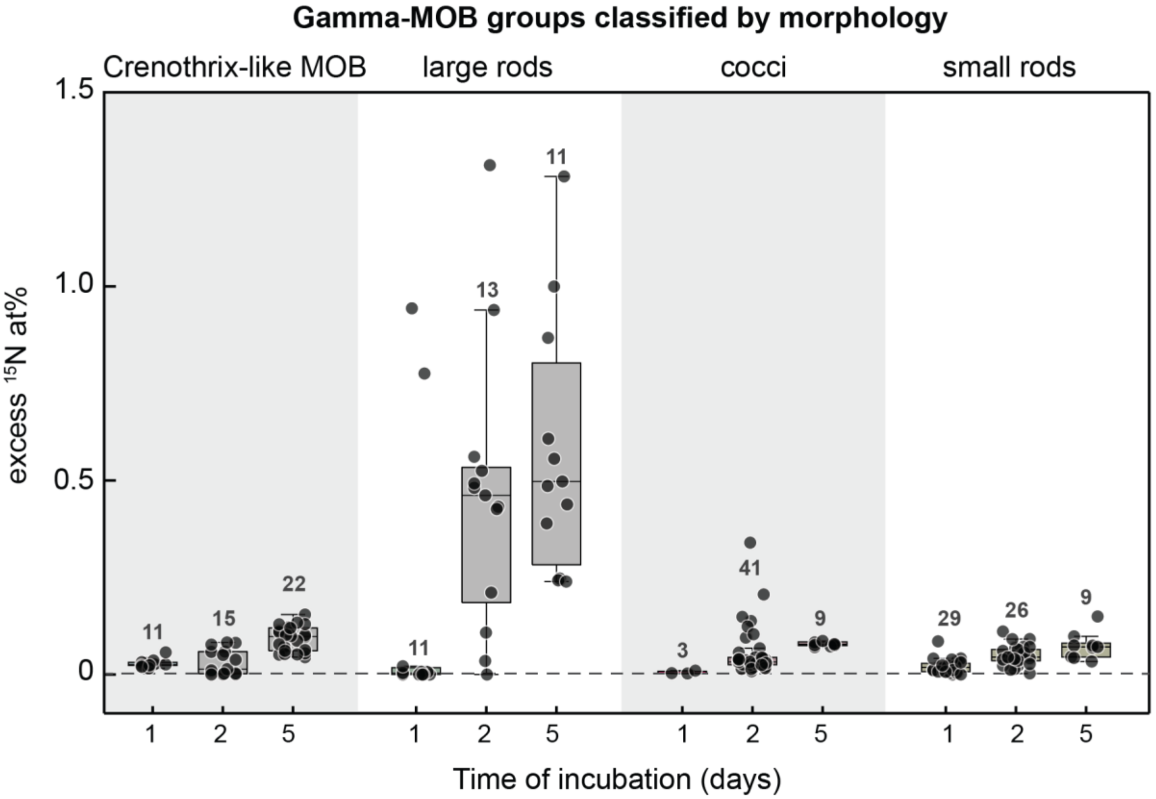

Figure S6. ^{15}N assimilation by gamma-MOB in anoxic, ^{15}N-nitrate amended incubations. ^{15}N at% values are given as excess values for each analyzed cell. The 0 value on the y-axis depicts the natural abundance (0.36%). Boxplots depict the 25-75% quantile range, with the center line representing the median (50% quantile) and whiskers representing the 5 and 95 percentile. The number of cells analyzed per category (n) is shown as scatter and indicated above each boxplot. Source data are provided as a Source Data file.

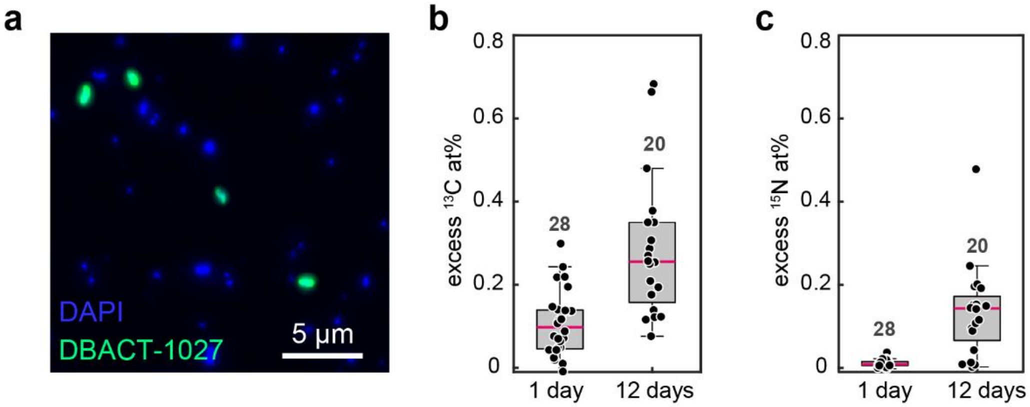

Figure S7. ^{13}C and ^{15}N assimilation of NC10 bacteria in anoxic incubations after 1 and 12 days. (**a**) CARD FISH image of NC10 bacteria in Lake Zug waters using probe DBACT-1027. (**b, c**) Excess ^{13}C and ^{15}N at% of NC10 bacteria after incubation with ^{13}C-labeled methane and ^{15}N-labeled nitrate after 1 and 12 days of anoxic incubation. ^{13}C and ^{15}N at% values are given as excess values for each analyzed cell. The 0 value on the y-axis depicts the natural abundance of ^{13}C (1.1%) and ^{15}N (0.36%). The number of cells analyzed per category (n) is shown as scatter and indicated above each boxplot. Boxplots depict the 25-75% quantile range, with the center line representing the median (50% quantile) and whiskers representing the 5 and 95 percentile. Source data are provided as a Source Data file.

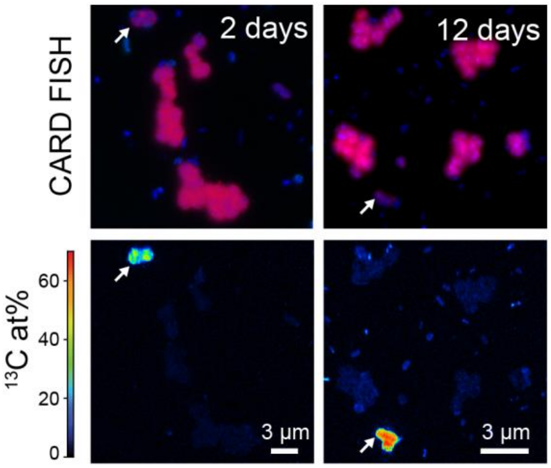

Figure S8. Negligible ^{13}C enrichment of cluster-forming coccoid gamma-MOB in anoxic incubations. Cluster-forming coccoid cells (stained by probes Mγ84 and Mγ705) (red) were not observed to have substantial ^{13}C enrichment in anoxic incubations as compared to their rod-shaped counterparts. Arrows mark rod-shaped cells that assimilated methane-derived carbon, as opposed to the cocci. Notably, after 12 days, also other, non-methanotrophic cells that were not targeted by the probe became ^{13}C enriched, presumably due to cross-feeding of methane-derived compounds from the MOB.

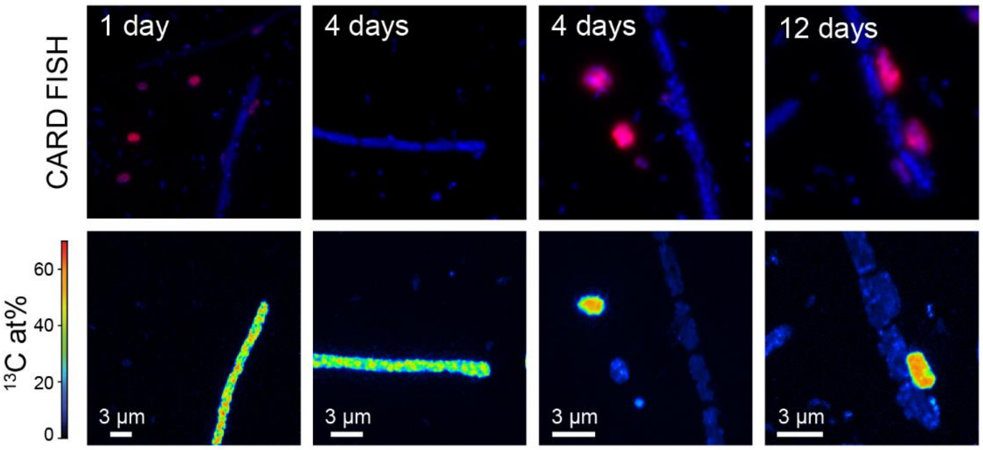

Figure S9. Variable ^{13}C enrichment of filamentous cells in anoxic incubations. Filamentous cells were observed to assimilate ^{13}C into their biomass but with large differences between individual filaments. For example, highly enriched filaments were observed on days 1 and 4, as compared to their counterparts observed on days 4 and 12.

Supplementary Tables

Table S1. Bulk methane oxidation and assimilation data. Methane oxidation (MO) rates to CO_2 and methane carbon (C) assimilation into biomass were determined through direct measurements obtained from stable isotope incubations. The percentages of methane-C assimilation were calculated based on these measurements.

Date	Water depth (m)	Condition	MO to CO_2 (μM d^{-1})	Methane-C assimilation into biomass (μM d^{-1})	Methane-C assimilation (%)
September 2017	123	Hypoxic	1.81	1.09	38
September 2017	135	Hypoxic	1.17	1.29	52
September 2017	160	Hypoxic	1.20	2.15	64
September 2017	123	Anoxic	0.07	0.02	22
September 2017	135	Anoxic	0.18	0.23	56
September 2017	160	Anoxic	0.06	0.09	60
October 2018	160	Hypoxic	0.59	0.51	46
October 2018	170	Hypoxic	4.30	1.42	25
October 2018	180	Hypoxic	4.05	2.10	34
October 2018	160	Anoxic	0.21	0.19	48
October 2018	170	Anoxic	0.72	0.43	37
October 2018	180	Anoxic	0.99	0.38	28
May 2019	175	Hypoxic	1.55	0.59	28
May 2019	181	Hypoxic	1.67	0.50	23
May 2019	185	Hypoxic	1.49	0.64	30
May 2019	175	Anoxic	0.12	0.06	33
May 2019	181	Anoxic	0.09	0.04	31
May 2019	185	Anoxic	0.12	0.05	29

Table S2. Growth rates, methane carbon assimilation, and cellular characteristics of morphologically distinct gamma-MOB populations in hypoxic and anoxic incubations.

	Growth rate based on ^{13}C assimilation (n) [(a)]	Average volume in μm^3 (n)	Excess ^{13}C at% $cell^{-1}$ d^{-1}	fmol ^{13}C $cell^{-1}$ d^{-1}
**Hypoxic incubations**				
Large rods	0.39 (26)	7.4±2.1 (13)	23.7	9.8
Small rods	0.34 (62)	1.6±1.0 (8)	20.8	4.2
Cocci	0.38 (21)	3.2±1.0 (25)	22.9	6.4
Filaments	0.49 (20)	55.3±45.1 (31)	28.98	30.1
**Anoxic incubations**				
Large rods	0.34 (12)	7.4±2.1 (13)	20.9	8.6
Small rods	0.01 (38)	1.6±1.0 (8)	1.1	0.2
Cocci	0.01 (36)	3.2±1.0 (25)	0.9	0.2
Filaments	0.03 (28)	55.3±45.1 (31)	5.9	2.4

[(a)] Based on average single cell ^{13}C enrichments determined by nanoSIMS analysis after 1 day of incubation, n = number of measured cells. To calculate ^{13}C assimilation per cell (in fmol ^{13}C $cell^{-1}$), first the carbon content per cell was calculated using the formula from Khachikyan et al. (2019) [31] taking into account the average biovolume per cell, multiplied with the excess ratio of $^{13}C/(^{12}C+^{13}C)$ derived from the nanoSIMS measurements. The cell-specific ^{13}C-based growth rates were calculated as described in Martínez-Pérez et al. (2016) [32].

Table S3. Experimental parameters used to calculate single cell-specific rates.

Experimental parameters	Hypoxic incubations 1 day	2 days	5 days	Anoxic incubations 1 day	2days	5 days	
Bulk $^{13}CO_2$ production [(a)]	0.66	2.74	4.53	0.22	0.52	0.83	µmol L^{-1}
Bulk ^{13}C assimilation [(a)]	2.52	4.61	9.68	0.76	1.04	1.62	µmol L^{-1}
Cell counts (large rods) [(b)]	2.99E+07	3.32E+07	7.94E+07	2.42E+07	2.48E+07	2.01E+07	cells L^{-1}
Cell counts (small rods) [(b)]	3.08E+08	5.10E+08	6.57E+08	2.09E+08	1.88E+08	1.22E+08	cells L^{-1}
Cell counts (cocci) [(b)]	2.58E+07	1.37E+07	2.58E+07	2.90E+07	1.42E+07	2.23E+06	cells L^{-1}
Cell counts (filaments) [(b)]	3.14E+06	4.78E+06	5.70E+06	3.69E+06	1.84E+06	1.95E+06	cells L^{-1}
^{13}C assimilation (large rods) [(c)]	9.76	14.68	18.03	8.64	13.60	18.99	fmol ^{13}C $cell^{-1}$
^{13}C assimilation (small rods) [(c)]	4.21	6.22	7.30	0.18	0.58	0.71	fmol ^{13}C $cell^{-1}$
^{13}C assimilation (cocci) [(c)]	6.40	8.49	9.79	0.17	0.33	1.74	fmol ^{13}C $cell^{-1}$
^{13}C assimilation (filaments) [(c)]	30.11	39.66	37.37	2.38	23.06	8.36	fmol ^{13}C $cell^{-1}$
Bulk ^{13}C assimilation by MOB [(d)]	1.85	3.97	6.69	0.26	0.49	0.49	µmol L^{-1}
**Single cell methane oxidation rate [(e)]**	**1.80**	**2.44**	**1.18**	**9.08**	**10.48**	**8.26**	**fmol $cell^{-1}$ d^{-1}**

[(a)] Based on direct measurement.

[(b)] Based on cell counts (CARD FISH).

[(c)] Calculated based on the single cell ^{13}C enrichment (nanoSIMS) and cell-specific carbon contents calculated using the formula from Khachikyan et al. (2019) [31].

[(d)] Calculated from gamma-MOB group-specific ^{13}C enrichments, where [(c)] was multiplied by the cell counts [(b)].

[(e)] Calculated from the bulk $^{13}CO_2$ production rate divided by the cell counts of all active gamma-MOB cells per group. Activity was inferred from nanoSIMS measurements.

Supplementary References

1 Wartiainen, I., Hestnes, A. G., McDonald, I. R. & Svenning, M. M. *Methylobacter tundripaludum* sp. nov., a methane-oxidizing bacterium from Arctic wetland soil on the Svalbard islands, Norway (78° N). International Journal of Systematic and Evolutionary Microbiology 56, 109-113, doi:https://doi.org/10.1099/ijs.0.63728-0 (2006).

2 van Grinsven, S., Sinninghe Damsté, J. S., Harrison, J., Polerecky, L. & Villanueva, L. Nitrate promotes the transfer of methane-derived carbon from the methanotroph *Methylobacter* sp. to the methylotroph *Methylotenera* sp. in eutrophic lake water. Limnology and Oceanography 66, 878-891, doi:https://doi.org/10.1002/lno.11648 (2021).

3 Khanongnuch, R., Mangayil, R., Svenning, M. M. & Rissanen, A. J. Characterization and genome analysis of a psychrophilic methanotroph representing a ubiquitous *Methylobacter* spp. cluster in boreal lake ecosystems. ISME Communications 2, 85, doi:10.1038/s43705-022-00172-x (2022).

4 Gulledge, J., Ahmad, A., Steudler, P. A., Pomerantz, W. J. & Cavanaugh, C. M. Family- and genus-level 16S rRNA-targeted oligonucleotide probes for ecological studies of methanotrophic bacteria. Applied and Environmental Microbiology 67, 4726-4733, doi:10.1128/AEM.67.10.4726-4733.2001 (2001).

5 Blees, J. et al. Micro-aerobic bacterial methane oxidation in the chemocline and anoxic water column of deep south-Alpine Lake Lugano (Switzerland). Limnology and Oceanography 59, 311-324, doi:https://doi.org/10.4319/lo.2014.59.2.0311 (2014).

6 Martinez-Cruz, K. et al. Anaerobic oxidation of methane by aerobic methanotrophs in sub-Arctic lake sediments. Sci Total Environ 607-608, 23-31, doi:10.1016/j.scitotenv.2017.06.187 (2017).

7 van Grinsven, S. et al. Methane oxidation in anoxic lake water stimulated by nitrate and sulfate addition. Environmental Microbiology 22, 766-782, doi:https://doi.org/10.1111/1462-2920.14886 (2020).

8 Hao, Q., Liu, F., Zhang, Y., Wang, O. & Xiao, L. *Methylobacter* accounts for strong aerobic methane oxidation in the Yellow River Delta with characteristics of a methane sink during the dry season. Science of the Total Environment 704, 135383, https://doi.org/10.1016/j.scitotenv.2019.135383 (2020).

9 Iguchi, H., Yurimoto, H. & Sakai, Y. *Methylovulum miyakonense* gen. nov., sp. nov., a type I methanotroph isolated from forest soil. Int J Syst Evol Microbiol 61, 810-815, doi:10.1099/ijs.0.019604-0 (2011).

10 Oshkin, I. Y. et al. *Methylovulum psychrotolerans* sp. nov., a cold-adapted methanotroph from low-temperature terrestrial environments, and emended description of the genus *Methylovulum*. International Journal of Systematic and Evolutionary Microbiology 66, 2417-2423, doi:https://doi.org/10.1099/ijsem.0.001046 (2016).

11 Mayr, M. J., Zimmermann, M., Guggenheim, C., Brand, A. & Bürgmann, H. Niche partitioning of methane-oxidizing bacteria along the oxygen–methane counter gradient of stratified lakes. The ISME Journal 14, 274-287, doi:10.1038/s41396-019-0515-8 (2020).

12 Rissanen, A. J. et al. Vertical stratification patterns of methanotrophs and their genetic controllers in water columns of oxygen-stratified boreal lakes. FEMS Microbiology Ecology 97, doi:10.1093/femsec/fiaa252 (2021).

13 Oswald, K. et al. Aerobic gammaproteobacterial methanotrophs mitigate methane emissions from oxic and anoxic lake waters. Limnology and Oceanography 61, S101-S118, doi:https://doi.org/10.1002/lno.10312 (2016).

14 Oswald, K. et al. *Crenothrix* are major methane consumers in stratified lakes. The ISME Journal 11, 2124-2140, doi:10.1038/ismej.2017.77 (2017).

15 Graf, J. S. et al. Bloom of a denitrifying methanotroph, '*Candidatus* Methylomirabilis limnetica', in a deep stratified lake. Environmental Microbiology 20, 2598-2614, doi:https://doi.org/10.1111/1462-2920.14285 (2018).

16 Graf, J. S. et al. Anaerobic endosymbiont generates energy for ciliate host by denitrification. Nature 591, 445-450, doi:10.1038/s41586-021-03297-6 (2021).

17 Gonzalez, J. M., Sherr, E. B. & Sherr, B. F. Size-selective grazing on bacteria by natural assemblages of estuarine flagellates and ciliates. Appl Environ Microbiol 56, 583-589, doi:10.1128/aem.56.3.583-589.1990 (1990).

18 Lee, S. et al. Methane-derived carbon flows into host-virus networks at different trophic levels in soil. Proceedings of the National Academy of Sciences 118, e2105124118, doi:10.1073/pnas.2105124118 (2021).

19 Naguib, M. Stoichiometry of methane oxidation in the methane-oxidizing strain M102 under the influence of various CH_4/O_2 mixtures. Zeitschrift für allgemeine Mikrobiologie 16, 437-444, doi:https://doi.org/10.1002/jobm.19760160604 (1976).

20 Ettwig, K. F. et al. Nitrite-driven anaerobic methane oxidation by oxygenic bacteria. Nature 464, 543-548, doi:10.1038/nature08883 (2010).

21 Kraft, B. et al. Oxygen and nitrogen production by an ammonia-oxidizing archaeon. Science 375, 97-100, doi:10.1126/science.abe6733 (2022).

22 Dershwitz, P. et al. Oxygen generation via water splitting by a novel biogenic metal ion-binding compound. Applied and Environmental Microbiology 87, e00286-00221, doi:10.1128/AEM.00286-21 (2021).

23 Raghoebarsing, A. A. et al. A microbial consortium couples anaerobic methane oxidation to denitrification. Nature 440, 918-921, doi:10.1038/nature04617 (2006).

24 Nauhaus, K., Treude, T., Boetius, A. & Krüger, M. Environmental regulation of the anaerobic oxidation of methane: a comparison of ANME-I and ANME-II communities. Environmental Microbiology 7, 98-106, doi:https://doi.org/10.1111/j.1462-2920.2004.00669.x (2005).

25 Ettwig, K. F., Alen, T. v., Pas-Schoonen, K. T. v. d., Jetten, M. S. M. & Strous, M. Enrichment and molecular detection of denitrifying methanotrophic bacteria of the NC10 phylum. Applied and Environmental Microbiology 75, 3656-3662, doi:10.1128/AEM.00067-09 (2009).

26 Milucka, J. et al. Zero-valent sulphur is a key intermediate in marine methane oxidation. Nature 491, 541-546, doi:10.1038/nature11656 (2012).

27 Reis, P. C. J., Thottathil, S. D. & Prairie, Y. T. The role of methanotrophy in the microbial carbon metabolism of temperate lakes. Nature Communications 13, 43, doi:10.1038/s41467-021-27718-2 (2022).

28 Nauhaus, K., Albrecht, M., Elvert, M., Boetius, A. & Widdel, F. In vitro cell growth of marine archaeal-bacterial consortia during anaerobic oxidation of methane with sulfate. Environmental Microbiology 9, 187-196, doi:https://doi.org/10.1111/j.1462-2920.2006.01127.x (2007).

29 Kitzinger, K. et al. Cyanate and urea are substrates for nitrification by Thaumarchaeota in the marine environment. Nature Microbiology 4, 234-243, doi:10.1038/s41564-018-0316-2 (2019).

30 Bristow, L. A. et al. Ammonium and nitrite oxidation at nanomolar oxygen concentrations in oxygen minimum zone waters. Proceedings of the National Academy of Sciences 113, 10601-10606, doi:10.1073/pnas.1600359113 (2016).

31 Khachikyan, A. et al. Direct cell mass measurements expand the role of small microorganisms in nature. Applied and Environmental Microbiology 85, e00493-00419, doi:10.1128/AEM.00493-19 (2019).

32 Martínez-Pérez, C. et al. The small unicellular diazotrophic symbiont, UCYN-A, is a key player in the marine nitrogen cycle. Nature Microbiology 1, 16163, doi:10.1038/nmicrobiol.2016.163 (2016).
