# Supplementary material for: Persistent activity of aerobic methane-oxidizing bacteria in anoxic lake waters due to metabolic versatility
Source: Nat Commun. 2024 Jun 21;15:5293. doi: 10.1038/s41467-024-49602-5 (PMC11192741; doi:10.1038/s41467-024-49602-5)
Supplement: Supplementary file 3 — Description of Additional Supplementary Files [file 41467_2024_49602_MOESM3_ESM.pdf]

Description of Additional Supplementary Files:

Supplementary Dataset 1: Methylococcales 16S rRNA gene phylogenetic tree, containing partial, metagenome assembled 16S rRNA gene sequences.

Supplementary Dataset 2: Statistics on methane oxidation and denitrification rates.

Supplementary Dataset 3: Metagenome-assembled 16S rRNA gene sequences from Lake Zug (in fasta format).

Supplementary Dataset 4: Raw phyloflash results.

Supplementary Dataset 5: List of genes related to methane oxidation, denitrification and fermentation analyzed in metatranscriptomic and metagenomic datasets.
