# Supplementary material for: Persistent activity of aerobic methane-oxidizing bacteria in anoxic lake waters due to metabolic versatility
Source: Nat Commun. 2024 Jun 21;15:5293. doi: 10.1038/s41467-024-49602-5 (PMC11192741; doi:10.1038/s41467-024-49602-5)
Supplement: Supplementary file 4 — Supplementary Dataset 1 [file 41467_2024_49602_MOESM4_ESM.pdf]

Supplementary Dataset 1
16S rRNA gene phylogeny

Genome-derived 16S rRNA gene sequences

16S rRNA gene sequences from metagenomes (this study)

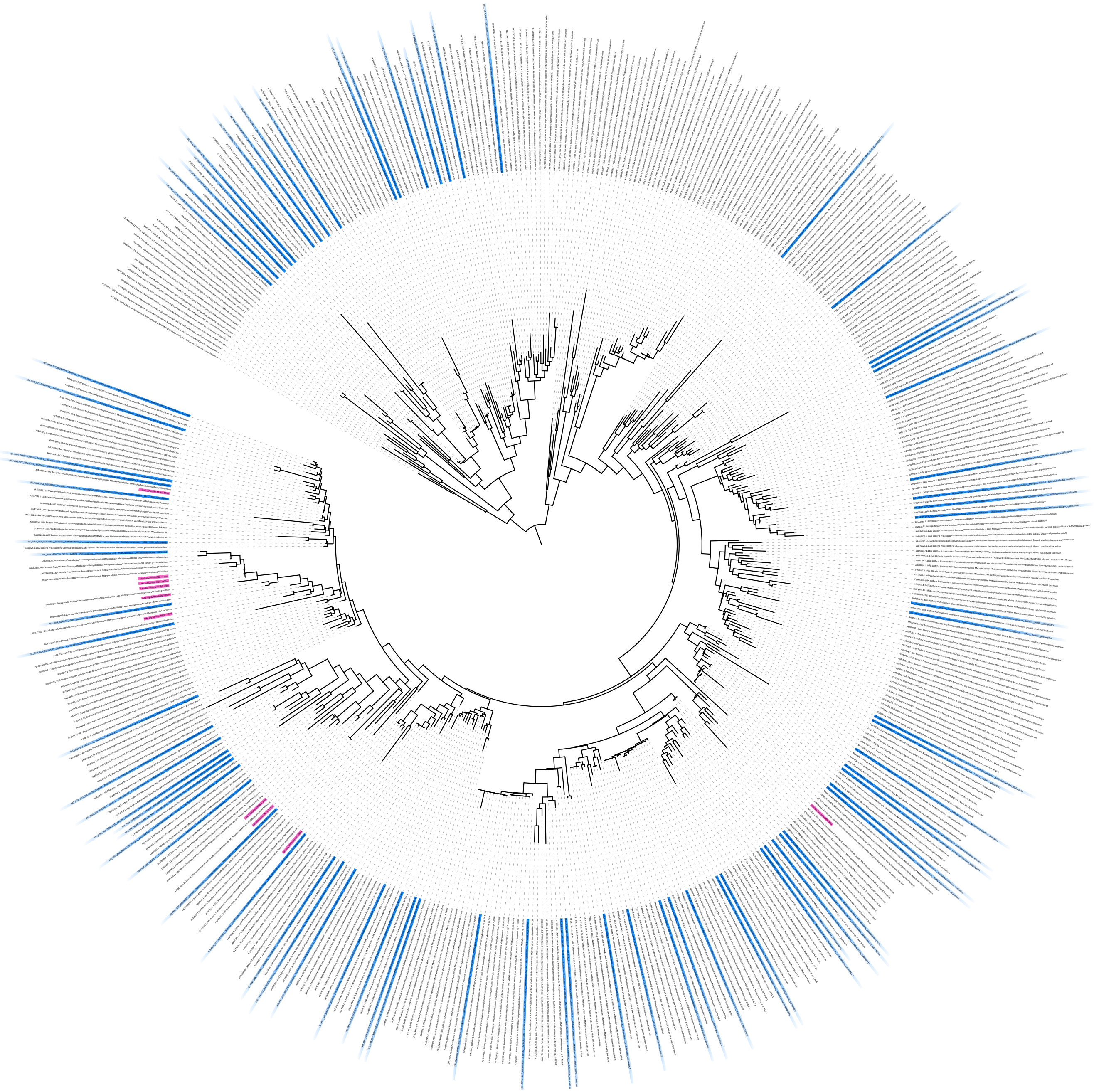

Tree scale: 0.1
